# Supplementary material for: Transdiagnostic compulsivity is associated with reduced reminder setting, only partially attributable to overconfidence
Source: eLife. 2025 May 29;13:RP98114. doi: 10.7554/eLife.98114 (PMC12122006; doi:10.7554/eLife.98114)
Supplement: Source code 2. [file elife-98114-code2.zip › remcomp05analysis_published.html]

remcomp05analysis\_published


Code 

- Show All Code
- Hide All Code

# remcomp05analysis\_published

#### Annika Boldt

#### 2025-03-24

- Load Data
- Exclusion
  Criteria
- Scale Data
- Demographics
- Sanity-check Hypotheses
  - H1
  - H2
  - H3
  - H4
- Key Hypotheses
  - H8 and H6
  - H5
  - H7
  - Moderation
    AD
- Other Analyses
  - Task Stats
  - Power
    Calculation
  - Parameter Recovery
    Psychometric Functions
  - Psychometric
    Reminder-Choice Function
  - Performance Differences
  - Stickiness Analysis
  - RT Analysis
  - Moderation/Mediation
    Analysis for OIP and AIP
  - Reminder
    Use in CIT

```
rm(list=ls(all=TRUE))

library(quickpsy)
library(pwr)
library(plyr)
library(effectsize)
library(diagram)
library(mediation)
library(lme4)
library(lmerTest)

# setwd(dirname(rstudioapi::getSourceEditorContext()$path))
```

## Load Data

This needs to run to load and preprocess the data from the server
(unfold by clicking on “Code” button).

```
# load workspace
load("remcomp05_published.RData")
replot = 0

ls()
```

```
## [1] "demodata" "mydata"   "npp"      "replot"
```

```
head(mydata)
```

```
##    sub gender age counterbal blockNum trialNum targetValue nHits reminderChoice
## 11   1      0  22          0        7        0           7     6              0
## 5    1      0  22          0        7        1           4     1              0
## 13   1      0  22          0        7        2           8     0              1
## 1    1      0  22          0        7        3           2     1              0
## 15   1      0  22          0        7        4           9     0              1
## 3    1      0  22          0        7        5           3     5              0
##    Ntargets points duration overwriteCond reminderActual circlesmoved
## 11        6     42    29527             1              1            6
## 5         6     52    14577             0              0            7
## 13        0     52     5073            NA              1            6
## 1         6     62    16151             0              0           21
## 15        0     62     5182            NA              1           22
## 3         6     77    22308             1              1            6
##    circlessteps circlesmovedagain circlesmovedearly
## 11          NaN                 0                 0
## 5          1.00                 0                 1
## 13          NaN                 0                 0
## 1          3.25                 0                 6
## 15         3.25                 1                 6
## 3           NaN                 0                 0
```

```
head(demodata)
```

```
##   pnum gender age edu counterbal gotwhatchosen       NPE remused accInternal
## 1    1      0  22   5          0        0.7500 0.1000000  0.3750    37.50000
## 2    2      1  38   7          0        0.7500 0.2500000  0.5000    45.83333
## 3    3      0  30   4          1        0.7500 0.0000000  0.2500    58.33333
## 4    4      0  31   6          0        0.6250 0.8000000  0.6875    54.16667
## 5    5      1  42   5          1        0.6875 0.2000000  0.3750    83.33333
## 6    6      0  22   4          0        0.7500 0.1111111  0.4375    66.66667
##   accInternal_odd accInternal_even accExternal accExternal_odd accExternal_even
## 1        50.00000         25.00000    95.83333       100.00000         91.66667
## 2        50.00000         41.66667    95.83333        91.66667        100.00000
## 3        58.33333         58.33333   100.00000       100.00000        100.00000
## 4        50.00000         58.33333   100.00000       100.00000        100.00000
## 5        83.33333         83.33333   100.00000       100.00000        100.00000
## 6        58.33333         75.00000   100.00000       100.00000        100.00000
##        OIP  OIP_odd OIP_even choice1 choice2 choice3 choice4 choice5 choice6
## 1 3.913043 5.000000 2.727273       0       0       0       0       0       0
## 2 4.782609 5.454545 4.166667       0       0       0       0       0       0
## 3 5.833333 5.833333 5.833333       0       0       0       0       0       0
## 4 5.416667 5.000000 5.833333       1       1       1       0       1       1
## 5 8.333333 8.333333 8.333333       0       0       0       0       0       0
## 6 6.666667 5.833333 7.500000       0       0       0       0       0       0
##   choice7 choice8 choice9 choice10 choice11 choice12 choice13 choice14 choice15
## 1       0       0       0        0        0        0        1        1        1
## 2       0       0       1        1        1        1        1        1        1
## 3       0       0       0        0        0        0        0        0        0
## 4       1       1       1        1        1        0        1        1        1
## 5       0       0       0        0        1        1        0        1        1
## 6       0       0       1        0        0        0        1        1        1
##   choice16      AIP     beta choiceCorrelation cj    rembias rembias_odd
## 1        1 7.512113 1.000000         0.7559289 62 -3.5990699 -2.51211341
## 2        1 5.500000 1.000000         0.8728716 10 -0.7173913 -0.04545455
## 3        0 9.000000 1.000000         0.0000000 70 -3.1666667 -3.16666667
## 4        0 2.000000 6.882019        -0.1747141 70  3.4166667  3.00000000
## 5        1 7.058878 1.042881         0.7356124 60  1.2744552  1.27445518
## 6        1 6.948278 1.040697         0.7356124 38 -0.2816115 -1.11494487
##   rembias_even  metabias metabias_odd metabias_even catchitem AES1 AES2 AES3
## 1   -4.7848407  24.50000     12.00000      37.00000         1    3    2    3
## 2   -1.3333333 -35.83333    -40.00000     -31.66667         1    2    3    2
## 3   -3.1666667  11.66667     11.66667      11.66667         1    3    3    3
## 4    3.8333333  15.83333     20.00000      11.66667         1    1    1    1
## 5    1.2744552 -23.33333    -23.33333     -23.33333         1    3    3    2
## 6    0.5517218 -28.66667    -20.33333     -37.00000         1    2    1    1
##   AES4 BIS1 BIS2 BIS3 BIS4 BIS5 BIS6 BIS7 BIS8 BIS9 BIS10 BIS11 STAI1 STAI2
## 1    3    3    3    3    3    2    3    2    2    2     2     2     3     3
## 2    2    3    1    2    2    2    3    1    3    1     1     1     2     2
## 3    2    4    1    3    4    1    2    1    3    2     3     2     3     3
## 4    1    1    2    3    2    2    3    2    2    2     2     2     2     1
## 5    3    2    2    2    2    2    2    2    2    3     3     3     2     3
## 6    1    1    2    1    1    1    1    4    1    1     3     3     1     1
##   STAI3 STAI4 STAI5 STAI6 STAI7 STAI8 STAI9 STAI10 STAI11 SDS1 SDS2 SDS3 SDS4
## 1     2     2     2     3     2     3     3      3      2    3    3    3    3
## 2     1     2     2     2     2     3     2      2      2    2    2    2    2
## 3     3     4     1     4     4     4     3      3      1    3    4    1    3
## 4     1     2     2     2     3     3     3      3      2    2    2    2    1
## 5     2     2     2     3     3     2     2      2      2    1    1    1    2
## 6     1     1     1     1     1     1     1      2      1    2    1    2    1
##   SDS5 SDS6 SDS7 SDS8 EAT1 EAT2 EAT3 EAT4 OCIR1 OCIR2 OCIR3 OCIR4 OCIR5 OCIR6
## 1    3    3    3    3    0    0    0    0     1     1     1     1     1     1
## 2    2    2    2    2    0    0    1    0     0     0     0     2     0     0
## 3    3    3    4    2    0    0    0    0     0     3     0     0     1     0
## 4    2    1    2    1    0    0    0    0     1     3     3     3     1     1
## 5    1    2    3    3    2    2    1    2     2     2     2     1     2     3
## 6    1    1    3    1    0    0    0    0     0     0     0     0     0     0
##   OCIR7 OCIR8 OCIR9 OCIR10 OCIR11 ICAR1 ICAR2 ICAR3 ICAR4 ICAR5 ICAR1_t ICAR2_t
## 1     3     2     2      1      2     4     6     4     2     2   58802   44941
## 2     0     0     0      0      0     1     2     2     2     3   63594   39384
## 3     1     1     2      0      0     4     2     1     1     7   53856   35133
## 4     0     1     0      0      0     4     2     3     5     2   14027   36787
## 5     2     1     2      2      1     4     1     4     1     8   36412   22751
## 6     0     0     0      1      0     4     2     2     2     6   19148   21431
##   ICAR3_t ICAR4_t ICAR5_t nBacknPrac nBackNonMatchCorr nBackMatchCorr
## 1  102445   60691   58779          1                75             16
## 2   13415   64752   45155          1                75             21
## 3   19438   48237   43541          1                80             12
## 4   79818   61785   84260          1                75             14
## 5   76552   26345    8173          1                73             19
## 6   25921   41176   83688          1                82             13
##   nBackNonMatchN nBackMatchN nBackdPrime nBackTime absremchosen OIP_clean
## 1             77          22    2.426575    103564            4  2.608696
## 2             75          24    3.559786    119241            8  4.347826
## 3             82          17    2.387514     99862            0  5.833333
## 4             79          20    2.084256    114688           13  5.833333
## 5             76          23    2.577768    143036            5  8.333333
## 6             86          13    3.431104    110576            5  7.500000
##   rembias_clean metabias_clean sumAES sumBIS sumSTAI sumSDS sumEAT sumOCIR
## 1    -4.9034178       12.00000     11     27      28     24      0      16
## 2    -1.1521739      -40.00000      9     20      22     16      1       2
## 3    -3.1666667       11.66667     11     26      33     23      0       8
## 4     3.8333333       20.00000      4     23      24     13      0      13
## 5     1.2744552      -23.33333     11     25      25     14      7      20
## 6     0.5517218      -20.33333      5     19      12     12      0       1
##   sumICAR points
## 1       5    231
## 2       1    249
## 3       1    260
## 4       2    274
## 5       2    320
## 6       2    304
```

One participant entered their age in the wrong format:

```
demodata$age[demodata$pnum=="559"] = 35
```

Next, we need to load the item weights (original source: https://osf.io/q3a6v).

```
# load item weights
itemweights = read.table("wise_weights.csv", header = TRUE, sep=",")

# scale the item scores 
qnForFacTrans=data.frame(scale(demodata[,which(names(demodata)=="AES1"):which(names(demodata)=="OCIR11")]))
# arrange item weights for transformation
itemWeightsArr<-data.frame(rbind(itemweights[46:49,],itemweights[31:41,],itemweights[9:19,],itemweights[1:8,],itemweights[42:45,],itemweights[20:30,]))
names(itemWeightsArr) <- c("item","AD","CIT")

# transform item scores into factor scores
transF1<-qnForFacTrans*t(itemWeightsArr[c("AD")])[col(qnForFacTrans)]
transF2<-qnForFacTrans*t(itemWeightsArr[c("CIT")])[col(qnForFacTrans)]
demodata<-data.frame(demodata,"AD"=rowSums(transF1),"CIT"=rowSums(transF2))
```

## Exclusion Criteria

```
excl1 = which((demodata$accExternal-demodata$accInternal)<0)
excl2 = which(demodata$accExternal<70)
excl3 = which(demodata$accInternal<10)


for(isub in 1:npp) {
  demodata$valdeccor[demodata$pnum==isub] = cor(mydata$targetValue[mydata$sub==isub],mydata$reminderChoice[mydata$sub==isub])
}
excl4 = which(demodata$valdeccor<0)


med_rem = median(demodata$rembias)
mad_rem = mad(demodata$rembias)
lower = med_rem - 3 * mad_rem
upper = med_rem + 3 * mad_rem
excl5a = c(which(demodata$rembias<lower),which(demodata$rembias>upper))

med_met = median(demodata$metabias)
mad_met = mad(demodata$metabias)
lower = med_met - 3 * mad_met
upper = med_met + 3 * mad_met
excl5b = c(which(demodata$metabias<lower),which(demodata$metabias>upper))

excl5 = c(excl5a,excl5b)


excl6 = which(demodata$catchitem!=1)

whichexcl = unique(c(excl1,excl2,excl3,excl4,excl5,excl6))
```

- *Hit rate higher on forced internal than forced external.*
  Based on this, we need to exclude 9 participants.
- *Below 70% accuracy on forced external trials.* Based on
  this, we need to exclude 22 participants.
- *Below 10% accuracy on forced internal trials.* Based on
  this, we need to exclude 3 participants.
- *Negative correlation between value and reminder choice (1=yes,
  0=no) as this would indicate participants did not understand the
  instructions.* Based on this, we need to exclude 40
  participants.
- *Participants who score lower or higher 3 times the median
  absolute deviation (MAD), calculated seperately based on both the
  reminder bias and the metacognitive bias.* Based on this, we need to
  exclude 0 participants.
- *Participants who have not answered with “Do not agree at all” to
  the catch item will be excluded.* Based on this, we need to exclude
  9 participants.

In total and taking into account that these criteria are not mutually
exclusive, 69 participants excluded.

Note that I copy-pasted text from the preregistration in italics
throughout this document.

```
if(replot) {
  quartz(width=8, height=6)
}

layout(1)
par(cex.main = 4.2, mar = c(4, 5, 2, 0), mgp = c(3, 1.0, 0), cex.lab = 1.6, font.lab = 1.6, cex.axis = 1.4, bty = "n", lwd=1, pch=19, las=1)
tjitter = rnorm(npp,1,0.08)
plot(tjitter,demodata$accInternal,type="n",xlim=c(0,3),ylim=c(0,100),xlab="",ylab="Accuarcy",axes=FALSE)
for(pnum in 1:npp) {
  if(demodata$accInternal[pnum]<10) {
    points(tjitter[pnum],demodata$accInternal[pnum],pch=19,col="red")
  } else {
    if (is.element(pnum,whichexcl)) {
      points(tjitter[pnum],demodata$accInternal[pnum],pch=19,col="red")
    } else {
      points(tjitter[pnum],demodata$accInternal[pnum],pch=19,col="black")
      }
    }
  if(demodata$accExternal[pnum]<70) {
    points(tjitter[pnum]+1,demodata$accExternal[pnum],pch=19,col="red")
  } else {
    if (is.element(pnum,whichexcl)) {
      points(tjitter[pnum]+1,demodata$accExternal[pnum],pch=19,col="red")
    } else {
      points(tjitter[pnum]+1,demodata$accExternal[pnum],pch=19,col="black")
      }  
    }
  if(demodata$accInternal[pnum]>demodata$accExternal[pnum]) {
    lines(c(tjitter[pnum],tjitter[pnum]+1),c(demodata$accInternal[pnum],demodata$accExternal[pnum]),col = alpha("black", 0.2))
  } else {
      lines(c(tjitter[pnum],tjitter[pnum]+1),c(demodata$accInternal[pnum],demodata$accExternal[pnum]),col = alpha("black", 0.2))
  }
}
lines(c(0.7,1.3),c(10,10),lty="dashed")
lines(c(1.7,2.3),c(70,70),lty="dashed")

axis(1, c(1,2), c("No Reminder", "Reminder"), lwd=2, mgp = c(3, 1.2, 0), cex.axis=1.6)
axis(2, c(0,25,50,75,100), c(0,25,50,75,100), lwd=2, mgp = c(3, 0.9, 0), cex.axis=1.4)

boxplot(demodata$accInternal[!is.element(demodata$pnum,whichexcl)], add=TRUE, col="darkgrey",
        at=0.5, axes=F, range=10)
boxplot(demodata$accExternal[!is.element(demodata$pnum,whichexcl)], add=TRUE, col="darkgrey",
        at=2.5, axes=F, range=10)
```

```
if(replot) {
  quartz.save("remcomp05_1.png", type="png", dpi=300)
  dev.off()
}
```

```
# reducing the sample by excluding identified participants

demodata_excl = subset(demodata,pnum==whichexcl[isub])
for(isub in 1:length(whichexcl)) {
  mydata = subset(mydata,sub!=whichexcl[isub])
  demodata_excl = rbind(demodata_excl,subset(demodata,pnum==whichexcl[isub]))
  demodata = subset(demodata,pnum!=whichexcl[isub])
}
oldsubs = demodata$pnum
for(isub in 1:nrow(demodata)) {
  mydata$sub[mydata$sub==oldsubs[isub]] = isub
}
demodata$pnum = 1:nrow(demodata)
demodata_excl$pnum = 1:nrow(demodata_excl)
npp = nrow(demodata)
npp_excl = nrow(demodata_excl)
```

This figure visualizes the exclusions shown in red. With 69 excluded
out of 669, this is a rate of 10.3%. This number excludes the 26
participants that were excluded for technical reasons. With them it
would be an exclusion rate of 13.7%.

## Scale Data

```
# scaling the demographics
demodata$gender <- factor(demodata$gender)
demodata$age.sc = scale(as.numeric(demodata$age))
demodata$sumICAR.sc = scale(demodata$sumICAR)
demodata$edu.sc = scale(demodata$edu)

# scaling the questionnaire scores
demodata$sumAES.sc = scale(demodata$sumAES) # apathy
demodata$sumBIS.sc = scale(demodata$sumBIS) # impulsivity
demodata$sumSTAI.sc = scale(demodata$sumSTAI) # trait anxiety
demodata$sumSDS.sc = scale(demodata$sumSDS) # depression
demodata$sumEAT.sc = scale(demodata$sumEAT) # eating disorder
demodata$sumOCIR.sc = scale(demodata$sumOCIR) # OCD

# scaling the factor scores
demodata$AD.sc = scale(demodata$AD)
demodata$CIT.sc = scale(demodata$CIT)

# scaling the 2-back task
demodata$nBackdPrime.sc = scale(demodata$nBackdPrime)

# scaling the metabias and rembias etc.
demodata$rembias.sc = scale(demodata$rembias)
demodata$rembias_clean.sc = scale(demodata$rembias_clean)
demodata$metabias.sc = scale(demodata$metabias)
demodata$metabias_clean.sc = scale(demodata$metabias_clean)
demodata$absremchosen.sc = scale(demodata$absremchosen)
demodata$AIP.sc = scale(demodata$AIP)
demodata$OIP.sc = scale(demodata$OIP)
demodata$cj.sc = scale(demodata$cj)
demodata$accInternal.sc = scale(demodata$accInternal)
demodata$accExternal.sc = scale(demodata$accExternal)
```

## Demographics

We included *N* = 600. In our remaining sample, 375 identified
as male, 218 as female and 7 as other. Participants were on average 32.9
years old (min = 18; max = 76).

## Sanity-check Hypotheses

### H1

*The reminder bias and metacognitive bias are negatively
correlated (one-sided test). This effect tests the above-mentioned link
between metacognition and cognitive offloading and constitutes the key
replication compared to previous studies. We will conduct this analysis
as in Gilbert et al. (2020) except for splitting the accuracy data to
avoid potentially inflating the correlation. More specifically, we sort
all trials into conditions: 1) 8 choice trials, 2) 4 trials in which
participants are forced to use reminders (forced external) and 3) 4
trials in which participants are forced to do the task using their own
memory (forced internal), having presented all conditions intermixed
throughout the study. Odd forced external trials and even forced
internal trials are used to calculate the reminder bias, whereas even
forced external trials and odd forced internal trials are used to
calculate the metacognitive bias. The resulting unconfounded biases will
subsequently be entered into a Pearson correlation analysis.*

```
cor.test(demodata$metabias_clean,demodata$rembias_clean,alternative="less")
```

```
## 
##  Pearson's product-moment correlation
## 
## data:  demodata$metabias_clean and demodata$rembias_clean
## t = -4.8918, df = 598, p-value = 6.43e-07
## alternative hypothesis: true correlation is less than 0
## 95 percent confidence interval:
##  -1.0000000 -0.1306598
## sample estimates:
##        cor 
## -0.1961549
```

```
# prepare some things for plotting
x <- demodata$metabias_clean
y <- demodata$rembias_clean
df <- data.frame(x = x, y = y)
model <- lm(y ~ x, data = df)
newx <- seq(min(df$x), max(df$x), length.out=100)
preds <- predict(model, newdata = data.frame(x=newx), interval = 'confidence')


if(replot) {
  quartz(width=8, height=6.5)
}

layout(1)
par(cex.main = 4.2, mar = c(5, 5, 2, 1), mgp = c(3, 1.0, 0), cex.lab = 1.6, font.lab = 1.6, cex.axis = 1.4, bty = "n", lwd=4, pch=19, las=1)
plot(demodata$metabias_clean,demodata$rembias_clean,type="n",xlab="Metacognitive Bias",ylab="Reminder Bias",xlim=c(-95,95),ylim=c(-7.5,7.5))
abline(model, lwd=1, lty="solid")
lines(newx, preds[ ,3], lwd=1, lty = 'dashed', col = 'black')
lines(newx, preds[ ,2], lwd=1, lty = 'dashed', col = 'black')
points(demodata$metabias_clean,demodata$rembias_clean,col = alpha("black", 0.3),cex=0.5)
```

```
if(replot) {
  quartz.save("remcomp05_2.png", type="png", dpi=300)
  dev.off()
}
```

### H2

*An excessive use of reminders (optimal indifference points
significantly higher than actual indifference points using a one-sided
paired t-test). For this analysis we do not need to use the odd/even
logic as inputs to the analysis are calculated from separate data points
anyways.*

In other words, we expect the reminder bias to be greater than
zero.

```
t.test(demodata$rembias,alternative="greater")
```

```
## 
##  One Sample t-test
## 
## data:  demodata$rembias
## t = 5.0808, df = 599, p-value = 2.514e-07
## alternative hypothesis: true mean is greater than 0
## 95 percent confidence interval:
##  0.3541956       Inf
## sample estimates:
## mean of x 
## 0.5241422
```

```
cohens_d(demodata$rembias,alternative="greater")$Cohens_d
```

```
## [1] 0.2074247
```

### H3

*Participants are underconfident in their own memory (expressed in
an average, negative metacognitive bias). This will be tested using a
one-sided, one-sample t-test ignoring the odd/even split of
trials.*

```
t.test(demodata$metabias,alternative="less")
```

```
## 
##  One Sample t-test
## 
## data:  demodata$metabias
## t = -3.0917, df = 599, p-value = 0.001041
## alternative hypothesis: true mean is less than 0
## 95 percent confidence interval:
##       -Inf -1.699891
## sample estimates:
## mean of x 
## -3.638889
```

```
cohens_d(demodata$metabias,alternative="less")$Cohens_d
```

```
## [1] -0.1262163
```

### H4

*Optimal and actual indifference points are positively correlated
(one-sided test; Spearman’s rho due to the data most likely being
distributed around the edges of the scale), suggesting participants who
benefited most from reminders were more likely to use them. Equally, for
this analysis we do not need to use the odd/even logic.*

```
cor.test(demodata$OIP,demodata$AIP,alternative="greater",method="spearman")
```

```
## Warning in cor.test.default(demodata$OIP, demodata$AIP, alternative =
## "greater", : Cannot compute exact p-value with ties
```

```
## 
##  Spearman's rank correlation rho
## 
## data:  demodata$OIP and demodata$AIP
## S = 23021742, p-value < 2.2e-16
## alternative hypothesis: true rho is greater than 0
## sample estimates:
##       rho 
## 0.3605054
```

## Key Hypotheses

### H8 and H6

*Negative link between AD factor and metacognitive bias (i.e. more
anxious-depressed individuals tend to be underconfident; e.g. Hoven et
al., 2019; Hypothesis H8a). This hypothesis is not a replication within
the context of this task and the test will therefore be carried out
two-sided. The metacognitive bias will be calculated from all trials
ignoring the odd/even logic used to test H1. The AD factor will be
calculated as in Seow & Gillan (2020) with the two differences that
we will use the reduced item set from Wise & Dolan (2020) and that
we will additionally include educational attainment as a covariate. The
regression model fit will be identical to the one used for H6. We
furthermore expect the effect to persist even if IQ is included as a
covariate (Hypothesis H8b).*

*We expect a significant link between the CIT factor and
metacognitive bias (Hypothesis H6a), expressed in a significant
predictor in the following regression model: metacognitive\_bias ~ AD +
CIT + age + gender + education Based on previous findings, both
directions of this effect are plausible. Studies with OCD patients have
commonly found underconfidence in OCD patients compared to healthy
controls (e.g. Hoven, Lebreton, Engelmann, Denys, Luigjes, & van
Holst, 2019); whereas several recent transdiagnostic studies with
healthy subjects have found the opposite (Rouault, Seow, Gillan &
Fleming, 2018; Seow & Gillan, 2020; Benwell, Mohr, Wallberg,
Kouadio, & Ince, 2022). The test will therefore be carried out
two-sided. The metacognitive bias will be calculated from all trials
ignoring the odd/even logic used to test H1. We plan to conduct the same
analysis based on raw confidence (percentage of circles participants
predicted they will remember; Hypothesis H6b). We furthermore expect the
effect to persist even if IQ is included as a covariate (Hypothesis
H6c).*

```
metabiasFac<-lm(metabias.sc ~ AD.sc + CIT.sc + age.sc + gender + edu.sc, data=demodata)
summary(metabiasFac)
```

```
## 
## Call:
## lm(formula = metabias.sc ~ AD.sc + CIT.sc + age.sc + gender + 
##     edu.sc, data = demodata)
## 
## Residuals:
##      Min       1Q   Median       3Q      Max 
## -2.89256 -0.72518  0.01676  0.73733  2.73890 
## 
## Coefficients:
##               Estimate Std. Error t value Pr(>|t|)    
## (Intercept)  0.0658816  0.0505745   1.303  0.19320    
## AD.sc       -0.2291842  0.0459234  -4.991 7.92e-07 ***
## CIT.sc       0.1450251  0.0465982   3.112  0.00195 ** 
## age.sc      -0.0228156  0.0418172  -0.546  0.58554    
## gender1     -0.1718940  0.0835092  -2.058  0.03999 *  
## gender2     -0.2937224  0.3785527  -0.776  0.43811    
## edu.sc      -0.0001835  0.0406895  -0.005  0.99640    
## ---
## Signif. codes:  0 '***' 0.001 '**' 0.01 '*' 0.05 '.' 0.1 ' ' 1
## 
## Residual standard error: 0.9783 on 593 degrees of freedom
## Multiple R-squared:  0.0526, Adjusted R-squared:  0.04301 
## F-statistic: 5.487 on 6 and 593 DF,  p-value: 1.529e-05
```

```
tbars = c(unlist(summary(metabiasFac))$coefficients2,
          unlist(summary(metabiasFac))$coefficients3)
tCI = array(NA,c(2,2))
tCI[,1] = confint(metabiasFac, 'AD.sc', level=0.95)
tCI[,2] = confint(metabiasFac, 'CIT.sc', level=0.95)


if(replot) {
  quartz(width=8, height=7)
}

layout(1)
par(cex.main = 2.0, mar = c(3, 5.5, 3, 0.5), mgp = c(3, 1, 0), cex.lab = 1.6, font.lab = 1.6, cex.axis = 1.4, bty = "n", lwd=2, pch=19, las=1)
mp = barplot(tbars, beside = TRUE, ylim=c(-0.35,0.30),col="white",names.arg=c("AD","CIT"),ylab=c(""),main="Associations with Metacognitive Bias",axes=FALSE,las=1,cex.names=1.8)
abline(h=0)
for (i in 1:2) {
    arrows(mp[i], tCI[1,i], mp[i], tCI[2,i], col ="black", code = 3, angle = 90, length = 0.1)
}
text(mp[1],tCI[1,1]-0.02,"***",cex=1.6)
text(mp[2],tCI[2,2]+0.02,"**",cex=1.6)
axis(2, c(-0.2,0,0.2), c(-0.2,0,0.2), cex.axis=1.8, lwd=2)
mtext(text="Standardised Beta",side=2, las=3, line=4.0, cex=1.8, at=0)
```

```
if(replot) {
  quartz.save("remcomp05_3.png", type="png", dpi=300)
  dev.off()
}


rawcjFac<-lm(cj.sc ~ AD.sc + CIT.sc + age.sc + gender + edu.sc, data=demodata)
summary(rawcjFac)
```

```
## 
## Call:
## lm(formula = cj.sc ~ AD.sc + CIT.sc + age.sc + gender + edu.sc, 
##     data = demodata)
## 
## Residuals:
##     Min      1Q  Median      3Q     Max 
## -2.5512 -0.7741  0.1695  0.7241  2.0939 
## 
## Coefficients:
##             Estimate Std. Error t value Pr(>|t|)    
## (Intercept)  0.08920    0.04921   1.813 0.070358 .  
## AD.sc       -0.28713    0.04468  -6.426 2.69e-10 ***
## CIT.sc       0.12496    0.04534   2.756 0.006025 ** 
## age.sc      -0.13999    0.04069  -3.441 0.000621 ***
## gender1     -0.23805    0.08125  -2.930 0.003522 ** 
## gender2     -0.23252    0.36831  -0.631 0.528078    
## edu.sc       0.04014    0.03959   1.014 0.311005    
## ---
## Signif. codes:  0 '***' 0.001 '**' 0.01 '*' 0.05 '.' 0.1 ' ' 1
## 
## Residual standard error: 0.9518 on 593 degrees of freedom
## Multiple R-squared:  0.1032, Adjusted R-squared:  0.09411 
## F-statistic: 11.37 on 6 and 593 DF,  p-value: 4.742e-12
```

```
metabiasFac_IQ1<-lm(metabias.sc ~ AD.sc + CIT.sc + age.sc + gender + edu.sc + sumICAR.sc, data=demodata)
summary(metabiasFac_IQ1)
```

```
## 
## Call:
## lm(formula = metabias.sc ~ AD.sc + CIT.sc + age.sc + gender + 
##     edu.sc + sumICAR.sc, data = demodata)
## 
## Residuals:
##      Min       1Q   Median       3Q      Max 
## -2.75447 -0.69390  0.04032  0.72298  2.57470 
## 
## Coefficients:
##             Estimate Std. Error t value Pr(>|t|)    
## (Intercept)  0.08356    0.04978   1.679   0.0937 .  
## AD.sc       -0.20254    0.04541  -4.460 9.81e-06 ***
## CIT.sc       0.11818    0.04607   2.565   0.0106 *  
## age.sc      -0.02727    0.04106  -0.664   0.5067    
## gender1     -0.21548    0.08246  -2.613   0.0092 ** 
## gender2     -0.45185    0.37301  -1.211   0.2262    
## edu.sc       0.03699    0.04067   0.909   0.3635    
## sumICAR.sc  -0.19647    0.04056  -4.844 1.63e-06 ***
## ---
## Signif. codes:  0 '***' 0.001 '**' 0.01 '*' 0.05 '.' 0.1 ' ' 1
## 
## Residual standard error: 0.9602 on 592 degrees of freedom
## Multiple R-squared:  0.08871,    Adjusted R-squared:  0.07794 
## F-statistic: 8.233 on 7 and 592 DF,  p-value: 1.345e-09
```

### H5

*Positive link between CIT factor and reminder bias (i.e. more
compulsive individuals tend to show a greater pro-offloading bias,
relative to the optimal strategy; Hypothesis H5a). This hypothesis is
not a replication within the context of this task and the test will
therefore be carried out two-sided. The reminder bias will be calculated
from all trials ignoring the odd/even logic used to test H1. The CIT
factor will be calculated as in Seow & Gillan (2020) with the two
differences that we will use the reduced item set from Wise & Dolan
(2020) and that we will additionally include educational attainment as a
covariate instead of IQ: reminder\_bias ~ AD + CIT + age + gender +
education We plan to conduct the same analysis based on the absolute
number of reminders (Hypothesis H5b) and the AIP (actual indifference
point; see Gilbert et al., 2020; Hypothesis H5c) We expect this effect
to persist even if working memory performance (d’ from the 2-back task)
is included as a covariate (Hypothesis H5d). We furthermore expect this
effect to persist even if IQ is included as a covariate (Hypothesis
H5e). It should be highlighted that the regression model includes both
the CIT and AD factors, to separate out the potentially competing
influences of these predictors. The same applies to all following
analyses.*

```
rembiasFac<-lm(rembias.sc ~ AD.sc + CIT.sc + age.sc + gender + edu.sc, data=demodata)
summary(rembiasFac)
```

```
## 
## Call:
## lm(formula = rembias.sc ~ AD.sc + CIT.sc + age.sc + gender + 
##     edu.sc, data = demodata)
## 
## Residuals:
##      Min       1Q   Median       3Q      Max 
## -2.85053 -0.58739  0.04409  0.64542  2.83744 
## 
## Coefficients:
##              Estimate Std. Error t value Pr(>|t|)   
## (Intercept) -0.012063   0.051056  -0.236  0.81330   
## AD.sc        0.067562   0.046361   1.457  0.14556   
## CIT.sc      -0.136969   0.047042  -2.912  0.00373 **
## age.sc       0.071330   0.042215   1.690  0.09161 . 
## gender1      0.004791   0.084304   0.057  0.95470   
## gender2      0.884807   0.382156   2.315  0.02094 * 
## edu.sc      -0.058212   0.041077  -1.417  0.15696   
## ---
## Signif. codes:  0 '***' 0.001 '**' 0.01 '*' 0.05 '.' 0.1 ' ' 1
## 
## Residual standard error: 0.9876 on 593 degrees of freedom
## Multiple R-squared:  0.03447,    Adjusted R-squared:  0.0247 
## F-statistic: 3.529 on 6 and 593 DF,  p-value: 0.001933
```

```
tbars = c(unlist(summary(rembiasFac))$coefficients2,
          unlist(summary(rembiasFac))$coefficients3)
tCI = array(NA,c(2,2))
tCI[,1] = confint(rembiasFac, 'AD.sc', level=0.95)
tCI[,2] = confint(rembiasFac, 'CIT.sc', level=0.95)


if(replot) {
  quartz(width=8, height=7)
}

layout(1)
par(cex.main = 2.0, mar = c(3, 5.5, 3, 0.5), mgp = c(3, 1, 0), cex.lab = 1.6, font.lab = 1.6, cex.axis = 1.4, bty = "n", lwd=2, pch=19, las=1)
mp = barplot(tbars, beside = TRUE, ylim=c(-0.35,0.3),col="white",names.arg=c("AD","CIT"),ylab=c(""),main="Associations with Reminder Bias",axes=FALSE,las=1,cex.names=1.8)
abline(h=0)
for (i in 1:2) {
    arrows(mp[i], tCI[1,i], mp[i], tCI[2,i], col ="black", code = 3, angle = 90, length = 0.1)
}
text(mp[2],tCI[1,2]-0.02,"**",cex=1.6)
axis(2, c(-0.2,0,0.2), c(-0.2,0,0.2), cex.axis=1.8, lwd=2)
mtext(text="Standardised Beta",side=2, las=3, line=4.0, cex=1.8, at=0)
```

```
if(replot) {
  quartz.save("remcomp05_4.png", type="png", dpi=300)
  dev.off()
}


rembiasFac_absrem<-lm(absremchosen.sc ~ AD.sc + CIT.sc + age.sc + gender + edu.sc, data=demodata)
summary(rembiasFac_absrem)
```

```
## 
## Call:
## lm(formula = absremchosen.sc ~ AD.sc + CIT.sc + age.sc + gender + 
##     edu.sc, data = demodata)
## 
## Residuals:
##      Min       1Q   Median       3Q      Max 
## -1.95627 -0.77554 -0.03998  0.74936  1.84608 
## 
## Coefficients:
##             Estimate Std. Error t value Pr(>|t|)    
## (Intercept) -0.03435    0.05037  -0.682   0.4955    
## AD.sc        0.06094    0.04574   1.332   0.1833    
## CIT.sc      -0.08992    0.04641  -1.937   0.0532 .  
## age.sc       0.18239    0.04165   4.379 1.41e-05 ***
## gender1      0.07114    0.08318   0.855   0.3928    
## gender2      0.72926    0.37706   1.934   0.0536 .  
## edu.sc      -0.10439    0.04053  -2.576   0.0102 *  
## ---
## Signif. codes:  0 '***' 0.001 '**' 0.01 '*' 0.05 '.' 0.1 ' ' 1
## 
## Residual standard error: 0.9744 on 593 degrees of freedom
## Multiple R-squared:  0.06007,    Adjusted R-squared:  0.05056 
## F-statistic: 6.316 on 6 and 593 DF,  p-value: 1.877e-06
```

```
rembiasFac_AIP<-lm(AIP.sc ~ AD.sc + CIT.sc + age.sc + gender + edu.sc, data=demodata)
summary(rembiasFac_AIP)
```

```
## 
## Call:
## lm(formula = AIP.sc ~ AD.sc + CIT.sc + age.sc + gender + edu.sc, 
##     data = demodata)
## 
## Residuals:
##      Min       1Q   Median       3Q      Max 
## -1.78386 -0.84452  0.05285  0.91513  1.84751 
## 
## Coefficients:
##             Estimate Std. Error t value Pr(>|t|)    
## (Intercept)  0.02246    0.05051   0.445   0.6568    
## AD.sc       -0.08060    0.04586  -1.757   0.0794 .  
## CIT.sc       0.10461    0.04654   2.248   0.0250 *  
## age.sc      -0.16513    0.04176  -3.954  8.6e-05 ***
## gender1     -0.03761    0.08340  -0.451   0.6522    
## gender2     -0.75359    0.37804  -1.993   0.0467 *  
## edu.sc       0.09117    0.04063   2.244   0.0252 *  
## ---
## Signif. codes:  0 '***' 0.001 '**' 0.01 '*' 0.05 '.' 0.1 ' ' 1
## 
## Residual standard error: 0.9769 on 593 degrees of freedom
## Multiple R-squared:  0.05515,    Adjusted R-squared:  0.04559 
## F-statistic: 5.768 on 6 and 593 DF,  p-value: 7.515e-06
```

```
rembiasFac_WM<-lm(rembias.sc ~ AD.sc + CIT.sc + age.sc + gender + edu.sc + nBackdPrime.sc, data=demodata)
summary(rembiasFac_WM)
```

```
## 
## Call:
## lm(formula = rembias.sc ~ AD.sc + CIT.sc + age.sc + gender + 
##     edu.sc + nBackdPrime.sc, data = demodata)
## 
## Residuals:
##      Min       1Q   Median       3Q      Max 
## -2.73095 -0.56856  0.01358  0.68126  2.88627 
## 
## Coefficients:
##                 Estimate Std. Error t value Pr(>|t|)  
## (Intercept)    -0.011489   0.050851  -0.226   0.8213  
## AD.sc           0.057100   0.046377   1.231   0.2187  
## CIT.sc         -0.121519   0.047288  -2.570   0.0104 *
## age.sc          0.074880   0.042071   1.780   0.0756 .
## gender1         0.004085   0.083965   0.049   0.9612  
## gender2         0.857530   0.380784   2.252   0.0247 *
## edu.sc         -0.063730   0.040975  -1.555   0.1204  
## nBackdPrime.sc  0.097968   0.040639   2.411   0.0162 *
## ---
## Signif. codes:  0 '***' 0.001 '**' 0.01 '*' 0.05 '.' 0.1 ' ' 1
## 
## Residual standard error: 0.9836 on 592 degrees of freedom
## Multiple R-squared:  0.04386,    Adjusted R-squared:  0.03255 
## F-statistic: 3.879 on 7 and 592 DF,  p-value: 0.0003832
```

```
rembiasFac_IQ1<-lm(rembias.sc ~ AD.sc + CIT.sc + age.sc + gender + edu.sc + sumICAR.sc, data=demodata)
summary(rembiasFac_IQ1)
```

```
## 
## Call:
## lm(formula = rembias.sc ~ AD.sc + CIT.sc + age.sc + gender + 
##     edu.sc + sumICAR.sc, data = demodata)
## 
## Residuals:
##      Min       1Q   Median       3Q      Max 
## -2.85613 -0.59479  0.03353  0.64688  2.85379 
## 
## Coefficients:
##              Estimate Std. Error t value Pr(>|t|)   
## (Intercept) -0.013257   0.051233  -0.259  0.79591   
## AD.sc        0.065764   0.046740   1.407  0.15995   
## CIT.sc      -0.135157   0.047422  -2.850  0.00452 **
## age.sc       0.071631   0.042258   1.695  0.09058 . 
## gender1      0.007732   0.084875   0.091  0.92744   
## gender2      0.895479   0.383920   2.332  0.02001 * 
## edu.sc      -0.060721   0.041860  -1.451  0.14743   
## sumICAR.sc   0.013260   0.041748   0.318  0.75088   
## ---
## Signif. codes:  0 '***' 0.001 '**' 0.01 '*' 0.05 '.' 0.1 ' ' 1
## 
## Residual standard error: 0.9883 on 592 degrees of freedom
## Multiple R-squared:  0.03464,    Adjusted R-squared:  0.02322 
## F-statistic: 3.034 on 7 and 592 DF,  p-value: 0.003835
```

### H7

*CIT acts as a moderator on the link between confidence and
offloading. In other words, we expect to find that the correlation
between the metacognitive and the reminder bias to be weakened in highly
compulsive individuals. We plan to analyse this by fitting a regression
model across participants. As stated in H1, the reminder bias should be
predicted by the metacognitive bias. In addition to the intercept and
the main effects of both metacognitive bias and compulsivity, we model
the moderation effect by adding an interaction between metacognitive
bias and compulsivity to the model and test whether its predictor is
significantly different from zero (Hypothesis H7a). We expect this
effect to persist even if working memory performance (d’ from the 2-back
task) is included as an additional covariate (Hypothesis H7b). We
furthermore expect the effect to persist even if IQ is included as a
covariate (Hypothesis H7c).*

```
remmetaFac<-lm(rembias_clean.sc ~ metabias_clean.sc * CIT.sc + AD.sc + age.sc + gender + edu.sc, data=demodata)
summary(remmetaFac)
```

```
## 
## Call:
## lm(formula = rembias_clean.sc ~ metabias_clean.sc * CIT.sc + 
##     AD.sc + age.sc + gender + edu.sc, data = demodata)
## 
## Residuals:
##      Min       1Q   Median       3Q      Max 
## -2.87183 -0.59588 -0.03255  0.68591  2.50464 
## 
## Coefficients:
##                           Estimate Std. Error t value Pr(>|t|)    
## (Intercept)              -0.012535   0.049880  -0.251  0.80167    
## metabias_clean.sc        -0.187715   0.040266  -4.662 3.88e-06 ***
## CIT.sc                   -0.099242   0.046281  -2.144  0.03241 *  
## AD.sc                     0.030648   0.046086   0.665  0.50630    
## age.sc                    0.132649   0.041220   3.218  0.00136 ** 
## gender1                   0.003187   0.082542   0.039  0.96922    
## gender2                   0.989609   0.373053   2.653  0.00820 ** 
## edu.sc                   -0.081516   0.040159  -2.030  0.04282 *  
## metabias_clean.sc:CIT.sc -0.007242   0.040184  -0.180  0.85703    
## ---
## Signif. codes:  0 '***' 0.001 '**' 0.01 '*' 0.05 '.' 0.1 ' ' 1
## 
## Residual standard error: 0.9638 on 591 degrees of freedom
## Multiple R-squared:  0.08343,    Adjusted R-squared:  0.07103 
## F-statistic: 6.725 on 8 and 591 DF,  p-value: 1.884e-08
```

```
remmetaFac_WM<-lm(rembias_clean.sc ~ metabias_clean.sc * CIT.sc + AD.sc + age.sc + gender + edu.sc + nBackdPrime.sc , data=demodata)
summary(remmetaFac_WM)
```

```
## 
## Call:
## lm(formula = rembias_clean.sc ~ metabias_clean.sc * CIT.sc + 
##     AD.sc + age.sc + gender + edu.sc + nBackdPrime.sc, data = demodata)
## 
## Residuals:
##      Min       1Q   Median       3Q      Max 
## -2.84700 -0.58706 -0.04578  0.67522  2.54488 
## 
## Coefficients:
##                           Estimate Std. Error t value Pr(>|t|)    
## (Intercept)              -0.012824   0.049762  -0.258  0.79672    
## metabias_clean.sc        -0.174163   0.040767  -4.272 2.26e-05 ***
## CIT.sc                   -0.088447   0.046502  -1.902  0.05766 .  
## AD.sc                     0.025240   0.046061   0.548  0.58392    
## age.sc                    0.135061   0.041142   3.283  0.00109 ** 
## gender1                   0.004865   0.082352   0.059  0.95291    
## gender2                   0.968693   0.372326   2.602  0.00951 ** 
## edu.sc                   -0.085476   0.040115  -2.131  0.03352 *  
## nBackdPrime.sc            0.078702   0.040348   1.951  0.05158 .  
## metabias_clean.sc:CIT.sc -0.010539   0.040125  -0.263  0.79291    
## ---
## Signif. codes:  0 '***' 0.001 '**' 0.01 '*' 0.05 '.' 0.1 ' ' 1
## 
## Residual standard error: 0.9616 on 590 degrees of freedom
## Multiple R-squared:  0.08931,    Adjusted R-squared:  0.07541 
## F-statistic: 6.429 on 9 and 590 DF,  p-value: 9.705e-09
```

```
remmetaFac_IQ1<-lm(rembias_clean.sc ~ metabias_clean.sc * CIT.sc + AD.sc + age.sc + gender + edu.sc + sumICAR.sc , data=demodata)
summary(remmetaFac_IQ1)
```

```
## 
## Call:
## lm(formula = rembias_clean.sc ~ metabias_clean.sc * CIT.sc + 
##     AD.sc + age.sc + gender + edu.sc + sumICAR.sc, data = demodata)
## 
## Residuals:
##      Min       1Q   Median       3Q      Max 
## -2.87403 -0.59664 -0.02616  0.67917  2.51198 
## 
## Coefficients:
##                           Estimate Std. Error t value Pr(>|t|)    
## (Intercept)              -0.013413   0.050086  -0.268  0.78894    
## metabias_clean.sc        -0.186326   0.040811  -4.566 6.06e-06 ***
## CIT.sc                   -0.098219   0.046561  -2.109  0.03532 *  
## AD.sc                     0.029740   0.046316   0.642  0.52105    
## age.sc                    0.132810   0.041260   3.219  0.00136 ** 
## gender1                   0.005362   0.083223   0.064  0.94865    
## gender2                   0.996943   0.374902   2.659  0.00805 ** 
## edu.sc                   -0.083175   0.040922  -2.033  0.04255 *  
## sumICAR.sc                0.008895   0.041269   0.216  0.82943    
## metabias_clean.sc:CIT.sc -0.007131   0.040220  -0.177  0.85934    
## ---
## Signif. codes:  0 '***' 0.001 '**' 0.01 '*' 0.05 '.' 0.1 ' ' 1
## 
## Residual standard error: 0.9646 on 590 degrees of freedom
## Multiple R-squared:  0.0835, Adjusted R-squared:  0.06952 
## F-statistic: 5.973 on 9 and 590 DF,  p-value: 5.041e-08
```

```
tdemodata = demodata
tdemodata$CIT.sc = as.numeric(tdemodata$CIT.sc)
tdemodata$metabias.sc = as.numeric(tdemodata$metabias.sc)
tdemodata$metabias_clean.sc = as.numeric(tdemodata$metabias_clean.sc)
tdemodata$gender = as.numeric(tdemodata$gender)

# need to rerun some models as mediation package does not accept the dummy coded gender variable

metabiasFac_new<-lm(metabias_clean.sc ~ AD.sc + CIT.sc + age.sc + gender + edu.sc, data=tdemodata)
summary(metabiasFac_new)
```

```
## 
## Call:
## lm(formula = metabias_clean.sc ~ AD.sc + CIT.sc + age.sc + gender + 
##     edu.sc, data = tdemodata)
## 
## Residuals:
##      Min       1Q   Median       3Q      Max 
## -2.91727 -0.68903  0.00366  0.70904  2.94086 
## 
## Coefficients:
##             Estimate Std. Error t value Pr(>|t|)    
## (Intercept)  0.19028    0.11661   1.632  0.10327    
## AD.sc       -0.21412    0.04590  -4.665 3.81e-06 ***
## CIT.sc       0.13434    0.04677   2.873  0.00422 ** 
## age.sc       0.02943    0.04195   0.701  0.48327    
## gender      -0.13722    0.07896  -1.738  0.08277 .  
## edu.sc      -0.02165    0.04086  -0.530  0.59638    
## ---
## Signif. codes:  0 '***' 0.001 '**' 0.01 '*' 0.05 '.' 0.1 ' ' 1
## 
## Residual standard error: 0.9825 on 594 degrees of freedom
## Multiple R-squared:  0.04282,    Adjusted R-squared:  0.03476 
## F-statistic: 5.314 on 5 and 594 DF,  p-value: 8.69e-05
```

```
remmetaFac_new<-lm(rembias_clean.sc ~ metabias_clean.sc * CIT.sc + AD.sc + age.sc + gender + edu.sc, data=tdemodata)
summary(remmetaFac_new)
```

```
## 
## Call:
## lm(formula = rembias_clean.sc ~ metabias_clean.sc * CIT.sc + 
##     AD.sc + age.sc + gender + edu.sc, data = tdemodata)
## 
## Residuals:
##      Min       1Q   Median       3Q      Max 
## -2.86497 -0.60056 -0.03881  0.69978  2.51934 
## 
## Coefficients:
##                          Estimate Std. Error t value Pr(>|t|)    
## (Intercept)              -0.10049    0.11517  -0.873  0.38326    
## metabias_clean.sc        -0.18574    0.04043  -4.594 5.33e-06 ***
## CIT.sc                   -0.09521    0.04645  -2.050  0.04084 *  
## AD.sc                     0.04222    0.04605   0.917  0.35964    
## age.sc                    0.12795    0.04136   3.094  0.00207 ** 
## gender                    0.07264    0.07802   0.931  0.35223    
## edu.sc                   -0.08094    0.04033  -2.007  0.04523 *  
## metabias_clean.sc:CIT.sc -0.01019    0.04034  -0.253  0.80064    
## ---
## Signif. codes:  0 '***' 0.001 '**' 0.01 '*' 0.05 '.' 0.1 ' ' 1
## 
## Residual standard error: 0.9681 on 592 degrees of freedom
## Multiple R-squared:  0.07382,    Adjusted R-squared:  0.06287 
## F-statistic: 6.741 on 7 and 592 DF,  p-value: 1.051e-07
```

```
remmetaFac_med = mediate(metabiasFac_new,remmetaFac_new, treat="CIT.sc", mediator="metabias_clean.sc", boot=T)
```

```
## Running nonparametric bootstrap
```

```
summary(remmetaFac_med)
```

```
## 
## Causal Mediation Analysis 
## 
## Nonparametric Bootstrap Confidence Intervals with the Percentile Method
## 
##                          Estimate 95% CI Lower 95% CI Upper p-value   
## ACME (control)            -0.0250      -0.0441        -0.01   0.002 **
## ACME (treated)            -0.0263      -0.0507        -0.01   0.002 **
## ADE (control)             -0.0953      -0.1975         0.00   0.054 . 
## ADE (treated)             -0.0967      -0.1975         0.00   0.052 . 
## Total Effect              -0.1216      -0.2256        -0.02   0.016 * 
## Prop. Mediated (control)   0.2052       0.0553         0.87   0.018 * 
## Prop. Mediated (treated)   0.2165       0.0391         0.87   0.018 * 
## ACME (average)            -0.0256      -0.0462        -0.01   0.002 **
## ADE (average)             -0.0960      -0.1979         0.00   0.054 . 
## Prop. Mediated (average)   0.2108       0.0576         0.84   0.018 * 
## ---
## Signif. codes:  0 '***' 0.001 '**' 0.01 '*' 0.05 '.' 0.1 ' ' 1
## 
## Sample Size Used: 600 
## 
## 
## Simulations: 1000
```

```
if(unlist(summary(metabiasFac))$coefficients24<0.001) {
  pvaldiag1 = paste0("'",round(unlist(summary(metabiasFac))$coefficients3,2),"***'")
} else if(unlist(summary(metabiasFac))$coefficients24<0.01) {
  pvaldiag1 = paste0("'",round(unlist(summary(metabiasFac))$coefficients3,2),"**'")
} else if(unlist(summary(metabiasFac))$coefficients24<0.05) {
  pvaldiag1 = paste0("'",round(unlist(summary(metabiasFac))$coefficients3,2),"*'")
} else if(unlist(summary(metabiasFac))$coefficients24<0.10) {
  pvaldiag1 = paste0("'",round(unlist(summary(metabiasFac))$coefficients3,2),".'")
} else {
  pvaldiag1 = paste0("'",round(unlist(summary(metabiasFac))$coefficients3,2),"'")
}

if(unlist(summary(remmetaFac))$coefficients29<0.001) {
  pvaldiag2 = paste0("'",round(unlist(summary(remmetaFac))$coefficients2,2),"***'")
} else if(unlist(summary(remmetaFac))$coefficients29<0.01) {
  pvaldiag2 = paste0("'",round(unlist(summary(remmetaFac))$coefficients2,2),"**'")
} else if(unlist(summary(remmetaFac))$coefficients29<0.05) {
  pvaldiag2 = paste0("'",round(unlist(summary(remmetaFac))$coefficients2,2),"*'")
} else if(unlist(summary(remmetaFac))$coefficients29<0.10) {
  pvaldiag2 = paste0("'",round(unlist(summary(remmetaFac))$coefficients2,2),".'")
} else {
  pvaldiag2 = paste0("'",round(unlist(summary(remmetaFac))$coefficients2,2),"'")
}

if(unlist(summary(rembiasFac))$coefficients24<0.001) {
  pvaldiag3 = paste0("'",round(unlist(summary(rembiasFac))$coefficients3,2),"***")
} else if(unlist(summary(rembiasFac))$coefficients24<0.01) {
  pvaldiag3 = paste0("'",round(unlist(summary(rembiasFac))$coefficients3,2),"**")
} else if(unlist(summary(rembiasFac))$coefficients24<0.05) {
  pvaldiag3 = paste0("'",round(unlist(summary(rembiasFac))$coefficients3,2),"*")
} else if(unlist(summary(rembiasFac))$coefficients24<0.10) {
  pvaldiag3 = paste0("'",round(unlist(summary(rembiasFac))$coefficients3,2),".")
} else {
  pvaldiag3 = paste0("'",round(unlist(summary(rembiasFac))$coefficients3,2))
}

if(unlist(summary(remmetaFac))$coefficients30<0.001) {
  pvaldiag4 = paste0(" (",round(unlist(summary(remmetaFac))$coefficients3,2),"0***)'")
} else if(unlist(summary(remmetaFac))$coefficients30<0.01) {
  pvaldiag4 = paste0(" (",round(unlist(summary(remmetaFac))$coefficients3,2),"0**)'")
} else if(unlist(summary(remmetaFac))$coefficients30<0.05) {
  pvaldiag4 = paste0(" (",round(unlist(summary(remmetaFac))$coefficients3,2),"0*)'")
} else if(unlist(summary(remmetaFac))$coefficients30<0.10) {
  pvaldiag4 = paste0(" (",round(unlist(summary(remmetaFac))$coefficients3,2),"0.)'")
} else {
  pvaldiag4 = paste0(" (",round(unlist(summary(remmetaFac))$coefficients3,2),"0)'")
}


if(replot) {
  quartz(width=8, height=7)
}

data <- c(0, pvaldiag1, 0,
          0, 0, 0, 
          pvaldiag2, paste0(pvaldiag3,pvaldiag4), 0)
M<- matrix (nrow=3, ncol=3, byrow = TRUE, data=data)
plot<- plotmat (M, pos=c(1,2), 
                name= c( "Metacognitive\nBias","CIT", "Reminder\nBias"), 
                box.type = "rect", box.size = 0.12, box.prop=0.5,  curve=0)
```

```
if(replot) {
  quartz.save("remcomp05_5.png", type="png", dpi=300)
  dev.off()
}
# Needs manual cropping for manuscript
```

### Moderation AD

```
remmetaFacAD<-lm(rembias_clean.sc ~ metabias_clean.sc * AD.sc + CIT.sc + age.sc + gender + edu.sc, data=demodata)
summary(remmetaFacAD)
```

```
## 
## Call:
## lm(formula = rembias_clean.sc ~ metabias_clean.sc * AD.sc + CIT.sc + 
##     age.sc + gender + edu.sc, data = demodata)
## 
## Residuals:
##     Min      1Q  Median      3Q     Max 
## -2.8595 -0.5926 -0.0369  0.7029  2.5158 
## 
## Coefficients:
##                          Estimate Std. Error t value Pr(>|t|)    
## (Intercept)             -0.018575   0.050257  -0.370  0.71182    
## metabias_clean.sc       -0.187046   0.040240  -4.648 4.13e-06 ***
## AD.sc                    0.031042   0.046047   0.674  0.50050    
## CIT.sc                  -0.099164   0.046195  -2.147  0.03223 *  
## age.sc                   0.130066   0.041286   3.150  0.00171 ** 
## gender1                  0.003506   0.082446   0.043  0.96610    
## gender2                  0.989126   0.372736   2.654  0.00818 ** 
## edu.sc                  -0.077127   0.040394  -1.909  0.05670 .  
## metabias_clean.sc:AD.sc -0.036423   0.038845  -0.938  0.34881    
## ---
## Signif. codes:  0 '***' 0.001 '**' 0.01 '*' 0.05 '.' 0.1 ' ' 1
## 
## Residual standard error: 0.9631 on 591 degrees of freedom
## Multiple R-squared:  0.08474,    Adjusted R-squared:  0.07235 
## F-statistic:  6.84 on 8 and 591 DF,  p-value: 1.29e-08
```

## Other Analyses

### Task Stats

On average, people got what they chose on 74.7% trials (SD = 5.8).
They used reminders on 49.9% trials (SD = 16.5). Average confidence was
55.6 (SD = 24.2).

```
layout(matrix(1:6, 3, 2, byrow = TRUE))
par(cex.main = 1.6, mar = c(5, 4, 2, 0), mgp = c(3, 1.0, 0), cex.lab = 1.1, font.lab = 1.1, cex.axis = 1.1, bty = "n", lwd=2, pch=19, las=1)

mean(demodata$gotwhatchosen)
```

```
## [1] 0.7470833
```

```
mean(demodata$remused)
```

```
## [1] 0.4986458
```

```
mean(demodata$accInternal)
```

```
## [1] 59.22222
```

```
mean(demodata$accExternal)
```

```
## [1] 96.0625
```

### Power Calculation

```
# Rouault et al. (2018)
rsq_full = 0.09700544 # adjusted r^2 from the model confMeanFactorReg
rsq_part = 0.05327108 # adjusted r^2 from the model without CIT
f2 = (rsq_full-rsq_part)/(1 - rsq_full)
pwr1 = ceiling(pwr.f2.test(u=6,f2=f2, sig.level=0.05, power=0.8)$v) + 6 + 1
pwr1
```

```
## [1] 288
```

```
# Seow & Gillan (2020)
rsq_full = 0.09041293 # adjusted r^2 from the model rembiasFac
rsq_part = 0.04677744 # adjusted r^2 from the model without CIT
f2 = (rsq_full-rsq_part)/(1 - rsq_full)
pwr2 = ceiling(pwr.f2.test(u=6, f2=f2, sig.level=0.05, power=0.8)$v) + 6 + 1
pwr2
```

```
## [1] 291
```

### Parameter Recovery Psychometric Functions

```
demodata$AIP_rec = NA
demodata$beta_rec = NA
lower_asymptote <- 0  # Minimum response probability
upper_asymptote <- 1  # Maximum response probability

psychometric_function <- function(x, mu, sigma, lower_asymptote, upper_asymptote) {
  lower_asymptote + (upper_asymptote - lower_asymptote) * pnorm(x, mean = mu, sd = sigma)
}

# Stimulus levels
stimulus_levels <- rep(seq(2, 9, length.out = 8),each=2)

for(isub in 1:npp) {
    # Psychometric function parameters
    alpha <- demodata$AIP[demodata$pnum==isub]         # Threshold
    beta <- demodata$beta[demodata$pnum==isub]         # Slope
    
    probabilities <- psychometric_function(stimulus_levels, alpha, beta, lower_asymptote, upper_asymptote)

    # Simulate choices based on probabilities
    choices <- sapply(probabilities, function(p) {
      if (runif(1) < p) 1 else 0
    })

    # Output the results
    tempres = data.frame(stimulus_levels, probabilities, choices)
    tempres$numbers = 1
    tempres$probabilities = NULL
    
    fit = quickpsy(tempres, stimulus_levels, choices, numbers, parini=list(c(2,9),c(1,500)))
    demodata$AIP_rec[isub] = fit$par$par[1]
    demodata$beta_rec[isub] = fit$par$par[2]    
    
}
```

```
## Warning: `group_by_()` was deprecated in dplyr 0.7.0.
## ℹ Please use `group_by()` instead.
## ℹ See vignette('programming') for more help
## ℹ The deprecated feature was likely used in the quickpsy package.
##   Please report the issue to the authors.
## This warning is displayed once every 8 hours.
## Call `lifecycle::last_lifecycle_warnings()` to see where this warning was
## generated.
```

```
cor.test(demodata$AIP,demodata$AIP_rec)
```

```
## 
##  Pearson's product-moment correlation
## 
## data:  demodata$AIP and demodata$AIP_rec
## t = 70.43, df = 598, p-value < 2.2e-16
## alternative hypothesis: true correlation is not equal to 0
## 95 percent confidence interval:
##  0.9353618 0.9526838
## sample estimates:
##       cor 
## 0.9446777
```

```
cor.test(demodata$beta,demodata$beta_rec)
```

```
## 
##  Pearson's product-moment correlation
## 
## data:  demodata$beta and demodata$beta_rec
## t = 5.8897, df = 598, p-value = 6.459e-09
## alternative hypothesis: true correlation is not equal to 0
## 95 percent confidence interval:
##  0.1570500 0.3084148
## sample estimates:
##       cor 
## 0.2341509
```

```
if(replot) {
  quartz(width=8, height=7)
}

layout(1)
par(cex.main = 4.2, mar = c(4, 5, 2, 0), mgp = c(3, 1.0, 0), cex.lab = 1.6, font.lab = 1.6, cex.axis = 1.4, bty = "n", lwd=1, pch=19, las=1)
plot(demodata$AIP,demodata$AIP_rec,type="n",xlim=c(2,9),ylim=c(2,9),xlab="AIP",ylab="Recovered AIP",axes=FALSE)
points(demodata$AIP,demodata$AIP_rec,pch=19,col="black")
lines(c(0,10),c(0,10),lty="dashed")
axis(1, 2:9, 2:9, lwd=2, mgp = c(3, 1.2, 0), cex.axis=1.6)
axis(2, 2:9, 2:9, lwd=2, mgp = c(3, 0.9, 0), cex.axis=1.4)
```

```
if(replot) {
  quartz.save("Appendix 1–figure 1.png", type="png", dpi=300)
  dev.off()
}
```

### Psychometric Reminder-Choice Function

Here I plot the psychometric functions individually:

```
ppnums = matrix(rep(c(1,20),30)+rep(seq(0,580,20),each=2),ncol=2,byrow=T)
x <- seq(2, 9, 0.1)

for(i in 1:30) {
  
  if(replot) {
    quartz(width=8, height=10)
  }
  
  layout(matrix(1:20, 5, 4, byrow = TRUE))
  par(cex.main = 1.4, mar = c(5, 4, 2, 0), mgp = c(3, 1.0, 0), cex.lab = 1.1, font.lab = 1.1, cex.axis = 1.1, bty = "n", lwd=1, pch=19, las=1)
  for(pnum in ppnums[i,1]:ppnums[i,2]) {
    plot(x, pnorm(x, mean = demodata$AIP[pnum], sd = demodata$beta[pnum]), col="black", type = "l", ylim = c(0, 1), xlab = "Target Value", ylab = "", main=paste0("Sub ",pnum))
    abline(v=demodata$AIP[pnum])
    offcurvetemp = ddply(mydata[mydata$sub==pnum,], .(targetValue), summarise, meanrem=mean(reminderChoice))
    points(offcurvetemp$targetValue,offcurvetemp$meanrem, pch = 19)
  }
  if(replot) {
  quartz.save(paste0("Appendix 1–figure ",i+1,".png"), type="png", dpi=300)
  dev.off()
  }
  
}
```

### Performance Differences

*We plan to investigate the relationship between CIT and AD
respectively with actual task performance (accuracy on trials in which
participants were not allowed to use a reminder).*

```
if(replot) {
  quartz(width=8, height=5)
}

layout(matrix(1:2, 1, 2, byrow = TRUE))
par(cex.main = 1.6, mar = c(4, 5, 2, 0), mgp = c(3, 1.0, 0), cex.lab = 1.6, font.lab = 1.6, cex.axis = 1.4, bty = "n", lwd=4, pch=19, las=1)

plot(demodata$CIT,demodata$accInternal,type="n",xlab="CIT",ylab="Internal Accuracy",
     xlim=c(-1.4,2.6),ylim=c(0,110), main="CIT")
points(demodata$CIT,demodata$accInternal,col="black",cex = 0.4)

plot(demodata$AD,demodata$accInternal,type="n",xlab="AD",ylab="Internal Accuracy",
     xlim=c(-1.8,2.2),ylim=c(0,110),main="AD")
points(demodata$AD,demodata$accInternal,col="black",cex = 0.4)
```

```
if(replot) {
  quartz.save("Appendix 1–figure 32.png", type="png", dpi=300)
  dev.off()
}
```

```
perfInFac<-lm(accInternal.sc ~ AD.sc + CIT.sc + age.sc + gender + edu.sc, data=demodata)
summary(perfInFac)
```

```
## 
## Call:
## lm(formula = accInternal.sc ~ AD.sc + CIT.sc + age.sc + gender + 
##     edu.sc, data = demodata)
## 
## Residuals:
##      Min       1Q   Median       3Q      Max 
## -2.07243 -0.73027 -0.09106  0.68445  2.37210 
## 
## Coefficients:
##             Estimate Std. Error t value Pr(>|t|)    
## (Intercept)  0.01406    0.05132   0.274 0.784179    
## AD.sc       -0.01838    0.04660  -0.394 0.693380    
## CIT.sc      -0.06308    0.04729  -1.334 0.182779    
## age.sc      -0.14855    0.04244  -3.500 0.000499 ***
## gender1     -0.04368    0.08475  -0.515 0.606478    
## gender2      0.15484    0.38417   0.403 0.687049    
## edu.sc       0.05315    0.04129   1.287 0.198513    
## ---
## Signif. codes:  0 '***' 0.001 '**' 0.01 '*' 0.05 '.' 0.1 ' ' 1
## 
## Residual standard error: 0.9928 on 593 degrees of freedom
## Multiple R-squared:  0.02427,    Adjusted R-squared:  0.0144 
## F-statistic: 2.459 on 6 and 593 DF,  p-value: 0.02342
```

### Stickiness Analysis

*We plan to analyse whether participants show a response
“stickiness” in their reminder use as reported by Scarampi & Gilbert
(2020) and whether this correlates with the CIT (e.g. Shahar et al.,
2021) and AD factors.*

```
stickyres_red = array(NA,c(npp,2))

for(isub in 1:npp) {
  
  datanow = subset(mydata,sub==isub)
  if(datanow$reminderChoice[1]!=datanow$reminderActual[1]) {
    stickyres_red[isub,1] = datanow$reminderActual[1]
    stickyres_red[isub,2] = length(which(datanow$reminderChoice[2:16]==stickyres_red[isub,1]))/15
  
  }
}

t.test(stickyres_red[,2],mu=0.5)
```

```
## 
##  One Sample t-test
## 
## data:  stickyres_red[, 2]
## t = -8.4326, df = 156, p-value = 2.13e-14
## alternative hypothesis: true mean is not equal to 0.5
## 95 percent confidence interval:
##  0.2733288 0.3593675
## sample estimates:
## mean of x 
## 0.3163482
```

```
cohens_d(stickyres_red[,2],mu=0.5)
```

```
## Warning: Missing values detected. NAs dropped.
```

```
## Cohen's d |         95% CI
## --------------------------
## -0.67     | [-0.85, -0.50]
## 
## - Deviation from a difference of 0.5.
```

```
demodata$stickyres_red = stickyres_red[,2]
demodata$stickyres_red.sc = scale(stickyres_red[,2])

persevFac<-lm(stickyres_red.sc ~ AD.sc + CIT.sc + age.sc + gender + edu.sc, data=demodata)
summary(persevFac)
```

```
## 
## Call:
## lm(formula = stickyres_red.sc ~ AD.sc + CIT.sc + age.sc + gender + 
##     edu.sc, data = demodata)
## 
## Residuals:
##      Min       1Q   Median       3Q      Max 
## -1.44508 -0.83983 -0.06436  0.68499  2.17073 
## 
## Coefficients:
##             Estimate Std. Error t value Pr(>|t|)  
## (Intercept)  0.01159    0.10489   0.110   0.9122  
## AD.sc       -0.07868    0.09315  -0.845   0.3997  
## CIT.sc       0.19989    0.09218   2.168   0.0317 *
## age.sc       0.08029    0.09090   0.883   0.3785  
## gender1     -0.09697    0.16737  -0.579   0.5632  
## gender2      0.33195    0.71890   0.462   0.6449  
## edu.sc       0.06796    0.08106   0.838   0.4032  
## ---
## Signif. codes:  0 '***' 0.001 '**' 0.01 '*' 0.05 '.' 0.1 ' ' 1
## 
## Residual standard error: 0.998 on 150 degrees of freedom
##   (443 observations deleted due to missingness)
## Multiple R-squared:  0.04232,    Adjusted R-squared:  0.004013 
## F-statistic: 1.105 on 6 and 150 DF,  p-value: 0.3622
```

### RT Analysis

```
datanow = mydata
datanow$CIT.sc = NA
for(isub in 1:npp) {
  datanow$CIT.sc[datanow$sub==isub] = demodata$CIT.sc[isub]
  datanow$AD.sc[datanow$sub==isub] = demodata$AD.sc[isub]
  datanow$age.sc[datanow$sub==isub] = demodata$age.sc[isub]
  datanow$gender[datanow$sub==isub] = demodata$gender[isub]
  datanow$edu.sc[datanow$sub==isub] = demodata$edu.sc[isub]
}
datanow$duration.sc = scale(datanow$duration)
datanow$reminderActual = as.factor(datanow$reminderActual)
datanow$gender = as.factor(datanow$gender)
datanow$circlesmoved.sc = scale(datanow$circlesmoved)
datanow$circlessteps.sc = scale(datanow$circlessteps)
datanow$circlesmovedagain.sc = scale(datanow$circlesmovedagain)
datanow$circlesmovedearly.sc = scale(datanow$circlesmovedearly)

RTnoremFac_lmer <- lmer(duration.sc ~ reminderActual * CIT.sc + AD.sc + 
                     reminderActual:AD.sc + age.sc + gender + edu.sc + (1 | sub),
                   data = datanow, REML = TRUE, 
                   lmerControl(optimizer = "Nelder_Mead"))
summary(RTnoremFac_lmer)
```

```
## Linear mixed model fit by REML. t-tests use Satterthwaite's method [
## lmerModLmerTest]
## Formula: 
## duration.sc ~ reminderActual * CIT.sc + AD.sc + reminderActual:AD.sc +  
##     age.sc + gender + edu.sc + (1 | sub)
##    Data: datanow
## Control: lmerControl(optimizer = "Nelder_Mead")
## 
## REML criterion at convergence: 26879.7
## 
## Scaled residuals: 
##     Min      1Q  Median      3Q     Max 
## -1.8877 -0.8346 -0.1818  0.6975  6.8803 
## 
## Random effects:
##  Groups   Name        Variance Std.Dev.
##  sub      (Intercept) 0.07019  0.2649  
##  Residual             0.91113  0.9545  
## Number of obs: 9600, groups:  sub, 600
## 
## Fixed effects:
##                          Estimate Std. Error         df t value Pr(>|t|)    
## (Intercept)            -7.268e-02  2.094e-02  9.522e+02  -3.470 0.000544 ***
## reminderActual1         7.670e-02  2.013e-02  9.564e+03   3.810 0.000140 ***
## CIT.sc                 -8.572e-03  2.037e-02  1.153e+03  -0.421 0.673921    
## AD.sc                  -4.195e-02  2.042e-02  1.228e+03  -2.054 0.040207 *  
## age.sc                  1.139e-01  1.526e-02  5.927e+02   7.467 2.94e-13 ***
## gender2                 9.956e-02  3.045e-02  5.914e+02   3.270 0.001140 ** 
## gender3                -4.542e-02  1.381e-01  5.917e+02  -0.329 0.742253    
## edu.sc                  9.434e-03  1.484e-02  5.917e+02   0.636 0.525164    
## reminderActual1:CIT.sc  7.654e-02  2.277e-02  9.580e+03   3.361 0.000780 ***
## reminderActual1:AD.sc  -1.869e-02  2.273e-02  9.577e+03  -0.822 0.410956    
## ---
## Signif. codes:  0 '***' 0.001 '**' 0.01 '*' 0.05 '.' 0.1 ' ' 1
## 
## Correlation of Fixed Effects:
##             (Intr) rmndA1 CIT.sc AD.sc  age.sc gendr2 gendr3 edu.sc rA1:CI
## remndrActl1 -0.475                                                        
## CIT.sc       0.005  0.012                                                 
## AD.sc        0.025 -0.009 -0.441                                          
## age.sc       0.048 -0.041  0.200 -0.013                                   
## gender2     -0.531 -0.007 -0.037 -0.012 -0.058                            
## gender3     -0.115 -0.013 -0.027 -0.092  0.022  0.084                     
## edu.sc      -0.006  0.025 -0.060  0.130 -0.105 -0.010 -0.008              
## rmndA1:CIT.  0.006  0.000 -0.552  0.250  0.000  0.027 -0.027 -0.017       
## rmndrA1:AD. -0.007 -0.001  0.255 -0.573  0.016 -0.008  0.000 -0.005 -0.460
```

```
circlesmovedagainFac_lmer <- lmer(circlesmovedagain.sc ~ reminderActual * CIT.sc + 
                     AD.sc + age.sc + gender + edu.sc + (1 | sub),
                   data = datanow, REML = TRUE, 
                   lmerControl(optimizer = "Nelder_Mead"))
summary(circlesmovedagainFac_lmer)
```

```
## Linear mixed model fit by REML. t-tests use Satterthwaite's method [
## lmerModLmerTest]
## Formula: circlesmovedagain.sc ~ reminderActual * CIT.sc + AD.sc + age.sc +  
##     gender + edu.sc + (1 | sub)
##    Data: datanow
## Control: lmerControl(optimizer = "Nelder_Mead")
## 
## REML criterion at convergence: 27245.7
## 
## Scaled residuals: 
##     Min      1Q  Median      3Q     Max 
## -0.9998 -0.1666 -0.1155 -0.0892 20.4488 
## 
## Random effects:
##  Groups   Name        Variance Std.Dev.
##  sub      (Intercept) 0.02582  0.1607  
##  Residual             0.97372  0.9868  
## Number of obs: 9600, groups:  sub, 600
## 
## Fixed effects:
##                          Estimate Std. Error         df t value Pr(>|t|)  
## (Intercept)             1.948e-02  1.829e-02  1.145e+03   1.065   0.2869  
## reminderActual1        -2.126e-02  2.053e-02  9.562e+03  -1.036   0.3003  
## CIT.sc                  3.107e-02  1.731e-02  1.245e+03   1.795   0.0730 .
## AD.sc                   1.642e-02  1.383e-02  5.933e+02   1.188   0.2354  
## age.sc                  5.615e-03  1.260e-02  5.954e+02   0.446   0.6561  
## gender2                -1.860e-02  2.515e-02  5.937e+02  -0.739   0.4599  
## gender3                -1.992e-01  1.140e-01  5.943e+02  -1.747   0.0812 .
## edu.sc                 -2.203e-03  1.226e-02  5.943e+02  -0.180   0.8574  
## reminderActual1:CIT.sc -1.342e-02  2.058e-02  9.509e+03  -0.652   0.5143  
## ---
## Signif. codes:  0 '***' 0.001 '**' 0.01 '*' 0.05 '.' 0.1 ' ' 1
## 
## Correlation of Fixed Effects:
##             (Intr) rmndA1 CIT.sc AD.sc  age.sc gendr2 gendr3 edu.sc
## remndrActl1 -0.554                                                 
## CIT.sc       0.003  0.015                                          
## AD.sc        0.027 -0.015 -0.345                                   
## age.sc       0.055 -0.050  0.188 -0.004                            
## gender2     -0.501 -0.008 -0.040 -0.021 -0.058                     
## gender3     -0.106 -0.016 -0.019 -0.112  0.022  0.083              
## edu.sc      -0.012  0.031 -0.051  0.155 -0.105 -0.010 -0.008       
## rmndA1:CIT.  0.004 -0.001 -0.586 -0.023  0.010  0.032 -0.037 -0.026
```

```
circlesstepsFac_lmer <- lmer(circlessteps.sc ~ reminderActual * CIT.sc + 
                     AD.sc + age.sc + gender + edu.sc + (1 | sub),
                   data = datanow, REML = TRUE, 
                   lmerControl(optimizer = "Nelder_Mead"))
summary(circlesstepsFac_lmer)
```

```
## Linear mixed model fit by REML. t-tests use Satterthwaite's method [
## lmerModLmerTest]
## Formula: circlessteps.sc ~ reminderActual * CIT.sc + AD.sc + age.sc +  
##     gender + edu.sc + (1 | sub)
##    Data: datanow
## Control: lmerControl(optimizer = "Nelder_Mead")
## 
## REML criterion at convergence: 6660
## 
## Scaled residuals: 
##     Min      1Q  Median      3Q     Max 
## -4.5718 -0.3172  0.1767  0.5046  2.8905 
## 
## Random effects:
##  Groups   Name        Variance Std.Dev.
##  sub      (Intercept) 0.324    0.5692  
##  Residual             0.609    0.7804  
## Number of obs: 2530, groups:  sub, 600
## 
## Fixed effects:
##                          Estimate Std. Error         df t value Pr(>|t|)    
## (Intercept)             6.107e-02  3.931e-02  8.869e+02   1.553    0.121    
## reminderActual1        -4.333e-02  3.422e-02  2.281e+03  -1.266    0.206    
## CIT.sc                 -5.924e-02  3.677e-02  9.295e+02  -1.611    0.107    
## AD.sc                  -2.750e-02  3.218e-02  6.215e+02  -0.855    0.393    
## age.sc                  1.481e-01  2.940e-02  6.303e+02   5.037  6.2e-07 ***
## gender2                -1.466e-03  5.855e-02  6.235e+02  -0.025    0.980    
## gender3                 2.303e-01  2.650e-01  6.187e+02   0.869    0.385    
## edu.sc                  2.771e-02  2.853e-02  6.234e+02   0.972    0.332    
## reminderActual1:CIT.sc -2.011e-02  3.423e-02  2.288e+03  -0.587    0.557    
## ---
## Signif. codes:  0 '***' 0.001 '**' 0.01 '*' 0.05 '.' 0.1 ' ' 1
## 
## Correlation of Fixed Effects:
##             (Intr) rmndA1 CIT.sc AD.sc  age.sc gendr2 gendr3 edu.sc
## remndrActl1 -0.432                                                 
## CIT.sc      -0.006  0.030                                          
## AD.sc        0.024 -0.009 -0.382                                   
## age.sc       0.049 -0.041  0.205 -0.005                            
## gender2     -0.544 -0.008 -0.025 -0.021 -0.058                     
## gender3     -0.118 -0.015 -0.042 -0.114  0.022  0.085              
## edu.sc      -0.002  0.021 -0.068  0.155 -0.107 -0.011 -0.006       
## rmndA1:CIT.  0.032 -0.030 -0.461 -0.019  0.016 -0.001 -0.007 -0.015
```

### Moderation/Mediation Analysis for OIP and AIP

The correlation between OIP and AIP (H4) expresses the compensatory
nature of reminders: people who need reminders more tend to be the ones
who use them more.

```
OIPAIPFacboth<-lm(AIP.sc ~ OIP.sc * CIT.sc + AD.sc + OIP.sc:AD.sc + age.sc + gender + edu.sc, data=demodata)
summary(OIPAIPFacboth)
```

```
## 
## Call:
## lm(formula = AIP.sc ~ OIP.sc * CIT.sc + AD.sc + OIP.sc:AD.sc + 
##     age.sc + gender + edu.sc, data = demodata)
## 
## Residuals:
##      Min       1Q   Median       3Q      Max 
## -2.08994 -0.71034  0.03956  0.72192  2.28145 
## 
## Coefficients:
##               Estimate Std. Error t value Pr(>|t|)    
## (Intercept)    0.01275    0.04734   0.269  0.78784    
## OIP.sc         0.34117    0.03788   9.007  < 2e-16 ***
## CIT.sc         0.11855    0.04360   2.719  0.00674 ** 
## AD.sc         -0.07672    0.04311  -1.780  0.07565 .  
## age.sc        -0.11557    0.03957  -2.920  0.00363 ** 
## gender1       -0.01152    0.07826  -0.147  0.88303    
## gender2       -0.80083    0.35455  -2.259  0.02427 *  
## edu.sc         0.07156    0.03822   1.872  0.06168 .  
## OIP.sc:CIT.sc -0.07722    0.04421  -1.747  0.08119 .  
## OIP.sc:AD.sc   0.01431    0.04182   0.342  0.73239    
## ---
## Signif. codes:  0 '***' 0.001 '**' 0.01 '*' 0.05 '.' 0.1 ' ' 1
## 
## Residual standard error: 0.9144 on 590 degrees of freedom
## Multiple R-squared:  0.1764, Adjusted R-squared:  0.1638 
## F-statistic: 14.04 on 9 and 590 DF,  p-value: < 2.2e-16
```

### Reminder Use in CIT

```
perfExFac<-lm(accExternal.sc ~ AD.sc + CIT.sc + age.sc + gender + edu.sc, data=demodata)
summary(perfExFac)
```

```
## 
## Call:
## lm(formula = accExternal.sc ~ AD.sc + CIT.sc + age.sc + gender + 
##     edu.sc, data = demodata)
## 
## Residuals:
##     Min      1Q  Median      3Q     Max 
## -4.6953 -0.2327  0.4854  0.6837  1.0845 
## 
## Coefficients:
##             Estimate Std. Error t value Pr(>|t|)  
## (Intercept)  0.01913    0.05148   0.372   0.7104  
## AD.sc        0.04442    0.04675   0.950   0.3423  
## CIT.sc      -0.11996    0.04743  -2.529   0.0117 *
## age.sc      -0.10567    0.04257  -2.482   0.0133 *
## gender1     -0.05468    0.08500  -0.643   0.5203  
## gender2      0.06353    0.38533   0.165   0.8691  
## edu.sc       0.03803    0.04142   0.918   0.3589  
## ---
## Signif. codes:  0 '***' 0.001 '**' 0.01 '*' 0.05 '.' 0.1 ' ' 1
## 
## Residual standard error: 0.9958 on 593 degrees of freedom
## Multiple R-squared:  0.01838,    Adjusted R-squared:  0.008448 
## F-statistic: 1.851 on 6 and 593 DF,  p-value: 0.08721
```
